# Factors associated with sexual dysfunction in patients with colorectal cancer in Iran: a cross-sectional study

**DOI:** 10.1038/s41598-024-55465-z

**Published:** 2024-02-28

**Authors:** Amirmohammad Dahouri, Mohammad Hassan Sahebihagh, Neda Gilani

**Affiliations:** 1 Departement of Community Health Nursing, Faculty of Nursing and Midwifery, Tabriz University of Medical Sciences, Tabriz, Iran; 2 Department of Statistics and Epidemiology, Faculty of Health, Tabriz University of Medical Sciences, Tabriz, Iran

**Keywords:** Colorectal neoplasms, Sexual dysfunctions, Demographic factors, Health-related quality of life, Cancer, Diseases, Gastroenterology, Health care, Medical research, Oncology, Risk factors

## Abstract

Sexual dysfunction is a prevalent issue among individuals diagnosed with colorectal cancer (CRC), significantly impacting their quality of life. However, limited research has explored the factors associated with sexual dysfunction in CRC patients in Iran. This cross-sectional study aimed to identify the demographic factors that may contribute to sexual dysfunction in this population. A cross-sectional study involving CRC patients was conducted from April 1, 2022, to May 1, 2022, in Tabriz, Iran. Ethical approvals were obtained, and convenience sampling was employed at outpatient chemotherapy centers in five Tabriz hospitals. Validated questionnaires, including participants characteristics form, the Female Sexual Function Index (FSFI) for females and International Index of Erectile Function (IIEF) for males, were utilized. Data were analyzed using IBM SPSS Statistics version 24, employing descriptive statistics and stepwise linear regression to assess association between mentioned factors and sexual function. Among 256 participants, 50.4% were males, 49.6% were females, and 80.5% were married. The predominant age range was 50–60 years. The study findings revealed a high prevalence of sexual dysfunction among both female (Mean ± SD: 10.91 ± 8.67, Min–Max: 3.20–33.00) and male (Mean ± SD: 27.64 ± 16.28, Min–Max: 11–62) CRC patients. Factors such as the presence of a colostomy for FSFI (P < 0.001), type of treatment received for both FSFI and IIEF (P < 0.001), type of housing for both FSFI and IIEF (P < 0.001), occupation for FSFI (P < 0.001), presence of other diseases for FSFI (P = 0.047), and time since the last chemotherapy session for FSFI (P = 0.018), Education for IIEF (P = 0.026), and Age for IIEF (P = 0.002) were identified as significant factors of sexual dysfunction. These demographic factors demonstrated varying effects on sexual function, underscoring the complexity of this issue. The results underscore the significance of addressing sexual health concerns in CRC patients and highlight the necessity for tailored interventions to enhance their overall well-being. Healthcare providers should recognize the influence of demographic factors on sexual function and contemplate integrating sexual health assessments and interventions into the care of CRC patients. Further research is needed to comprehend better the underlying mechanisms and devise effective strategies for managing sexual dysfunction in this population.

## Introduction

Cancer encompasses a diverse range of diseases characterized by uncontrolled cellular proliferation and growth, posing a significant global health challenge and standing as the second leading cause of mortality in the United States^1,2^. Among the various types of cancer, colorectal cancer (CRC) ranks as the third most diagnosed cancer worldwide, with a staggering 1,849,518 reported cases in 2018, accounting for 10.2% of all cancers^3^. The incidence of CRC is projected to increase substantially, with an estimated 3.2 million new cases and 1.6 million deaths expected by 2040^4^. Notably, Iran has witnessed a notable rise in CRC incidence over the past 25 years, ranking it as the fourth most common cancer in the country, third most common in Iranian women, and fifth most common in Iranian men^5,6^. Tragically, CRC claims approximately 30,000 lives in Iran annually^7,8^. The surge in CRC cases can be attributed to factors such as increased life expectancy, lifestyle changes, and advancements in diagnostic and therapeutic approaches^9^.

The diagnosis of colon cancer profoundly impacts patients’ physical, emotional, and social aspects of life^10,11^. It represents a pivotal moment of rupture and psychological turmoil for patients, constituting a traumatic experience that significantly affects their overall quality of life^12^. Sexual dysfunction, encompassing a range of disorders characterized by clinically significant impairments in sexual response or pleasure, is a prevalent issue affecting approximately 43% of women and 31% of men^13–15^. Among the consequences of cancer, sexual dysfunction stands out as a distressing and common condition that often persists or worsens over time if left untreated^16,17^. Poor sexual functioning and low sexual satisfaction are risk factors associated with a diminished quality of life^18^. Notably, sexual dysfunction is widely acknowledged as one of the most prevalent long-term effects of cancer treatments^17,19,20^.

Recent studies have shed light on the sexual health of CRC patients and survivors, exploring the factors contributing to sexual dysfunction. Treatment-related factors such as extensive surgeries, invasive procedures, and radiotherapy have been identified as increasing the risk of sexual dysfunction^21^. Even the presence of a temporary or permanent ostomy has been associated with sexual challenges^22–24^. The impact of chemotherapy on sexual function is more challenging to assess, as it is often combined with surgery, making it difficult to differentiate their respective effects on patients' sexuality^21^. While most studies have focused on rectal cancer due to the direct impact of mutilating surgeries and radiotherapy on sexual function, evidence suggests that sexual disorders also occur in cases of colon cancer^21,25,26^. Even though patients with colon cancer may undergo treatments with less direct impact on their genital sphere, the disease itself and chemotherapy regimens significantly affect their overall quality of life^27^.

Healthcare providers involved in the care and management of CRC patients should consider sexual function and the multifaceted factors influencing it^28,29^. Early identification of patients at risk of sexual dysfunction enables timely interventions aimed at improving their well-being^30^. Furthermore, the influence of ethnicity, culture, gender, and socioeconomic status can introduce fundamental variations in the factors impacting sexual function^31–34^. Despite the high prevalence and increasing trend of CRC in Iran, coupled with the critical importance of sexual function, there remains a significant gap in organized studies exploring this complex interplay. The existing literature lacks a comprehensive investigation into the related factors associated with sexual dysfunction specifically in colorectal cancer patients in Iran. This cross-sectional study aims to fill this notable research gap by examining and elucidating the unique determinants of sexual dysfunction in this specific population.

## Methods

### Ethical consideration

This study adhered to strict ethical principles, obtaining all necessary approvals and permissions. Approval was granted by the Research Council and Research Vice-Chancellor of the Faculty of Nursing and Midwifery at Tabriz University, as well as the Research Vice-Chancellor of Tabriz University of Medical Sciences. The regional ethics committee provided approval under reference number IR.TBZMED.REC.1401.046. Participants were fully informed about the research objectives and invited to participate voluntarily. Confidentiality was ensured by using a coding system to anonymize participants' identities. At the chemotherapy outpatient departments, eligible individuals who expressed interest were invited to participate in the study and complete the research questionnaire. The researcher personally introduced themselves to the patients, providing a clear and detailed explanation of the research objectives. The voluntary nature of participation and the strict confidentiality of participants’ information were emphasized. The questionnaires were then distributed to the patients, and the researcher remained present throughout the completion process to provide assistance, address inquiries or concerns, and ensure accurate responses. Prior to starting the questionnaire, a concise guide was provided to ensure proper completion. The completed questionnaires were collected during the same session. Proper citation and referencing were employed to respect intellectual property rights, and research findings were shared with participants upon request. These ethical considerations were implemented to protect participants’ rights, maintain confidentiality, and uphold the integrity of the research.

### Study design

A cross-sectional study was conducted from April 1, 2022, to May 1, 2022, with the target population consisting of patients diagnosed with colon and rectal cancer. The sampling process did not consider the presence or absence of a colostomy or the permanence of the bag. All samples were recruited from outpatient chemotherapy centers. Convenience sampling was employed, and the researcher approached five hospitals in Tabriz, including Shahid Madani, Shahid Ghazi, Alinasab, Shahriar, and Valiasr. Qualified and interested individuals were invited to participate in the study by completing a research questionnaire. The researcher obtained permission from the hospital managers and administered the questionnaires to the patients without interrupting their treatment process. This study design aimed to ensure minimal disruption to patients' care and treatment while adhering to ethical considerations.

### Sample size calculation

The determination of the sample size in our study involved careful consideration of the independent factors, which amounted to 22. Following “Green’s rule of thumb,” which recommends a sample size of at least 50 + (8 * the number of predictors), we calculated that a total sample size of 226 would be appropriate^35^. This calculation took into account a significance level (α) of 0.05 and a desired power of 0.8. To ensure the detection of medium effect sizes (0.14 for small effects, 0.39 for medium effects, and 0.59 for large effects), we incorporated a conservative 10% allowance for potential participant dropout, resulting in a minimum sample size of 251 (226 * 0.9). Statistics Kingdom, a reliable tool, was utilized for determining the sample size^36^. The sample size determination in this study adhered to established academic guidelines and statistical considerations.

### Data collection

In this study, three questionnaires were used as follows:

#### Demographics and characteristics of the respondents

In this research, a questionnaire specifically designed by the researchers was utilized to evaluate the demographic and clinical attributes of the participants. The primary objective of the questionnaire was to gather comprehensive data on a variety of demographic and clinical variables.

#### Women's Sexual Performance Index (FSFI) Questionnaire

To assess the sexual function of female participants, the researchers employed the Female Sexual Function Index (FSFI). The FSFI questionnaire comprises 19 questions that evaluate six domains of sexual function: desire, arousal, moisture, orgasm, satisfaction, and pain. Participants rated their responses on a Likert scale. The total score was calculated by summing the scores from each domain. It is worth noting that the reliability and validity of the FSFI questionnaire have been previously established in studies conducted in Iran^37–40^.

#### International Index of Erectile Function in Men Questionnaire (IIEF)

In assessing the sexual performance of male participants, the researchers employed the International Index of Erectile Function (IIEF) Questionnaire. This questionnaire comprises 15 questions that evaluate different dimensions of erectile function, categorized into five subscales. It is important to note that it is worth mentioning that the reliability and validity of the IIEF questionnaire have been validated in previous studies conducted in Iran^41,42^.

### Data abstraction

#### Inclusion criteria

A confirmed diagnosis of CRC by an oncologist, the presence of a colostomy bag (whether permanent or temporary), effective communication skills, willingness to participate in the study, age above 30 years, referral for outpatient chemotherapy, adequate knowledge about their illness and treatment, active involvement in sexual activity, and a history of sexual function prior to the onset of the disease.

#### Non-inclusion criteria

**Figure 1:**
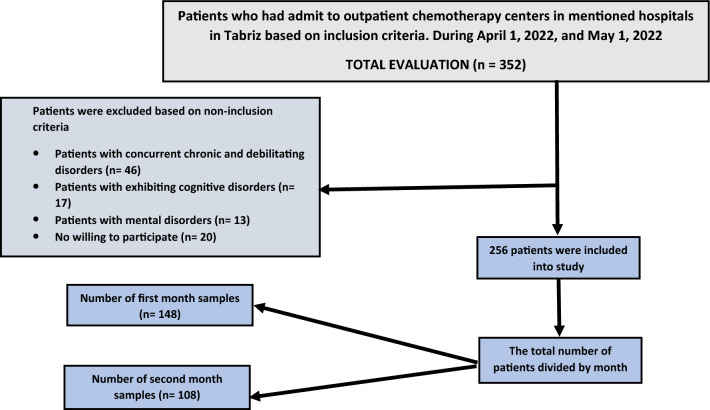
Consort flow diagram (inclusion/non-inclusion criteria).

Participants with concurrent chronic and debilitating diseases, including diabetes, kidney diseases, or any organ defects that could potentially affect sexual function, were not included in the study. Likewise, individuals who self-reported or were reported by their companions to have cognitive disorders, such as Alzheimer's disease, were not part of the participant pool. Known mental disorders, as self-reported by participants or documented in their medical records, also served as additional non-inclusion criteria. Additionally, individuals who expressed unwillingness to participate were not considered to ensure a more homogeneous participant group and minimize potential confounding factors that could impact the study outcomes. It is imperative to emphasize that Fig. 1 delineates the sampling flow diagram, furnishing a thorough representation of the participant selection process.

### Data analyzes

The data analysis was conducted using IBM SPSS Statistics version 24. Frequency and percentage distributions were employed to explore the characteristics of the study participants. Descriptive statistics, such as mean and standard deviation, were used to summarize variables with normal distributions. The normality of variable distributions was assessed using the Kolmogorov–Smirnov test, along with Skewness and Kurtosis indices. A significance level of 0.05 was chosen for all statistical tests performed in this study. To examine the potential predictive influence of the variables, a stepwise linear regression model was employed. All variables, including those with multiple categories (transformed into dummy variables), were entered into the regression analysis. The variables that demonstrated the most significant predictive effects were selected for subsequent statistical analysis. Notably, the dependent variable in this analysis was the total score of sexual function. It is imperative to emphasize that Fig. 1 delineates the sampling flow diagram, furnishing a thorough representation of the participant selection process.

### Ethics approval and consent to participate

Ethical approval for the study was granted by Tabriz University of Medical Sciences with ID: IR.TBZMED.REC.1401.046. (the research ethics committees certificate file has been uploaded in the supplementary section). Informed consent was obtained from all participants. There were no under 16 participants in this research. All methods were carried out in accordance with relevant guidelines and regulations.

## Results

Table 1 offers a summary of the study participants' characteristics. It is essential to highlight that information regarding cancer stage was not incorporated into the questionnaire covering participant characteristics.

**Table 1:** Distribution of frequency and percentage of individual characteristics of samples.

Variable	Classes	*N* (valid percent)
Age	30–40	67 (26.2)
	40–50	46 (18.0)
	50–60	95 (37.1)
	More than 60	48 (18.8)
Sex	Male	129 (50.4)
	Female	127 (49.6)
Marital status	Single	22 (8.6)
	Married	206 (80.5)
	Divorced and widowed	28 (10.9)
Education	Under diploma	50 (19.5)
	Diploma	73 (28.5)
	Bachelor	81 (31.6)
	Post graduate	52 (20.3)
Occupation	Employed	166 (64.8)
	Unemployed	90 (35.2)
Income adequacy	Income equals expenditure	138 (53.9)
	Income more than expenditure	42 (16.4)
	Income less than expenditure	76 (29.7)
Having insurance	Yes	228 (89.1)
	No	28 (10.9)
Location	City	240 (93.8)
	Village	16 (6.3)
Housing type	Personal	228 (89.1)
	Rent	28 (10.9)
Type of treatment	Only chemotherapy	81 (31.6)
	Chemotherapy-radiotherapy-surgery	98 (38.3)
	Chemotherapy-surgery	77 (30.1)
Time of last chemotherapy (week)	< 5	232 (90.6)
	≥ 5–10	24 (9.4)
	Mean (SD)	3.10 (4.04)
Family history	Positive	121 (47.3)
	Negative	135 (52.7)
Metastasis	Yes	149 (58.2)
	No	107 (41.8)
Number of chemotherapy courses (number)	< 10	137 (53.5)
	≥ 10–20	84 (32.8)
	≥ 20–30	35 (13.7)
	Mean (SD)	9.34 (6.98)
Another disease besides cancer	Yes	106 (41.4)
	No	150 (58.6)
Time of last surgery (month)	1–10 ≥	223 (87.1)
	10–20 and more	33 (12.9)
	Mean (SD)	6.11 (5.52)
Exercise (hour/week)	≤ 10	229 (89.5)
	10–20 and more	27 (10.5)
	Mean (SD)	3.42 (3.84)
Sexually active before the disease	Active	251 (98)
	Not active	5 (2)
Weight (kg)	45–65	63 (24.6)
	≥ 65–85	126 (49.2)
	≥ 85–105	67 (26.2)
	Mean (SD)	74.71 (14.42)
Height (cm)	70–130	3 (1.2)
	≥ 130–192	253 (98.8)
	Mean (SD)	169.34 (13.48)
Body mass index (kg/m^2^)	< 18.5	12 (4.7)
	≥ 18.5–25	123 (48)
	≥ 25–30	67 (26.2)
	≥ 30	54 (21.1)
	Mean (SD)	25.88 (4.74)
Having colostomy	With	127 (49.6)
	Without	129 (50.4)

Table 2 displays the scores achieved by the participants across various domains of the FSFI, as well as the total score. Based on the indicated cut-off points in the table, it is evident that all female participants exhibited poor performance of sexual functioning.

**Table 2:** The mean and standard deviation of FSFI (n = 127).

Variable	Cut off points	Mean (SD)	Min–Max
Sexual desire	Poor performance (≤ 3/3)	2.19 (1.24)	1.20–4.80
Arousal	Poor performance (≤ 4/3)	2.05 (1.13)	1.20–9.00
Lubrication	Poor performance (≤ 4/.3)	1.47 (1.80)	0.00–4.80
Orgasm	Poor performance (≤ 4/.3)	1.51 (1.86)	0.00–5.60
Satisfaction	Poor performance (≤ 8/3)	2.08 (1.46)	0.80–5.20
Sexual pain	Poor performance (≤ 8/3)	1.59 (1.89)	0.00–4.80
Total score	Poor performance (≤ 28)	10.91 (8.67)	3.20–33.00

Table 3 presents the scores achieved by male participants across various domains of the IIEF, as well as the total score. Based on the obtained scores and the specified cut-off points in the table, it is evident that all male participants exhibited “Moderate Dysfunction” levels in terms of their IIEF scores.

**Table 3:** The mean and standard deviation of IIEF (n = 129).

Variable	Cut off points	N (%)	Mean (SD)	Min–Max	Normalized mean (SD)
Erectile function	Severe disfunction (1–6)	39 (30.2)	10.98 (6.09)	2–26	36.59 (20.31)
	Moderate disfunction (7–12)	49 (38.0)			
	Mild to moderate disfunction (13–18)	23 (17.8)			
	Mild disfunction (19–24)	15 (11.6)			
	No disfunction (25–30)	3 (2.3)			
Orgasm function	Severe disfunction (0–2)	56 (43.4)	3.86 (2.03)	2–8	38.60 (20.33)
	Moderate disfunction (3–4)	35 (27.1)			
	Mild to moderate disfunction (5–6)	23 (17.8)			
	Mild disfunction (7–8)	15 (11.6)			
	No disfunction (9–10)	0 (0)			
Sexual desire	Severe disfunction (= 2)	56 (43.4)	3.86 (2.03)	2–8	23.26 (25.42)
	Moderate disfunction (3–4)	35 (27.1)			
	Mild to moderate disfunction (5–6)	23 (17.8)			
	Mild disfunction (7–8)	15 (11.6)			
	No disfunction (9–10)	0 (0)			
Satisfaction with sexual contact	Severe disfunction (0–3)	64 (49.6)	4.62 (4.93)	0–13	30.80 (32.91)
	Moderate disfunction (4–6)	11 (8.5)			
	Mild to moderate disfunction (7–9)	25 (19.4)			
	Mild disfunction (10–12)	27 (20.9)			
	No disfunction (13–15)	2 (1.6)			
Comprehensive satisfaction	Severe disfunction (= 2)	53 (41.1)	4.33 (2.50)	2–10	29.07 (31.25)
	Moderate disfunction (3–4)	35 (27.1)			
	Mild to moderate disfunction (5–6)	14 (10.9)			
	Mild disfunction (7–8)	18 (14.0)			
	No disfunction (9–10)	9 (7.0)			
Total score	Severe disfunction (6–20)	63 (48.8)	27.64 (16.27)	11–62	33.30 (22.92)
	Moderate disfunction (21–32)	21 (16.3)			
	Mild to moderate disfunction (33–45)	19 (14.7)			
	Mild disfunction (46–58)	21 (16.3)			
	No disfunction (59–75)	5 (3.9)			

Table 4 presents the coefficients derived from the step-by-step regression model, encompassing the 22 variables included in the analysis. Among these variables, six were found to exert the most significant potential impact on the FSFI. These six variables exhibited statistically significant potential effects, as indicated by their standardized beta values. Notably, the “Housing Type” variable demonstrated the greatest potential effect, with a 95% confidence interval of (3.96, − 12.73) and a significant p-value of less than 0.001.

**Table 4:** Results from stepwise multiple regression for total score of FSFI.

Factors	Β (95% CI)	Beta*	P-value
Having colostomy (with)	7.13 (4.63, 9.63)	0.41	< 0.001
Type of treatment (chemotherapy-surgery)	− 6.08 (− 8.61, − 3.55)	− 0.35	< 0.001
Housing type (rent)	8.35 (3.96, − 12.73)	0.30	< 0.001
Occupation (employed)	− 5.50 (− 8.28, − 2.72)	− 0.31	< 0.001
Another disease besides cancer (yes)	3.01 (0.5, 4.97)	0.17	0.047
Time of last chemotherapy (5–10 weeks or more)	− 5.24 (− 9.58, − 0.90)	− 0.20	0.018

*Standardized beta coefficient.

Table 5 displays the coefficients obtained from the step-by-step regression model, involving the 22 variables included in the analysis. Among these variables, four variables were found to have the most significant potential impact on the IIEF. These four variables exhibited statistically significant potential effects, as evidenced by their significant standardized beta values. Notably, the variable “Housing Type” exerted the greatest potential effect, with a 95% confidence interval of (− 33.87, − 10.50) and a significant p-value of less than 0.001.

**Table 5:** Results from stepwise multiple regression for total score of IIEF.

Factors	Β (95% CI)	Beta*	P-value
Education (bachelor and post-graduate)	− 11.09 (− 20.85, − 1.33)	− 0.18	0.026
Housing type (personal)	− 22.18 (− 33.87, − 10.50)	− 0.30	< 0.001
Age (more than 40)	17.07 (6.47, 27.68)	0.27	0.002
Type of treatment (chemotherapy-surgery)	− 17.86 (− 27.42, − 8.29)	− 0.38	< 0.001

*Standardized beta coefficient.

## Discussion

Scholars and healthcare professionals have demonstrated a keen interest in examining the quality of life among individuals affected by cancer for several decades^43^. However, investigations into the sexual well-being of these patients have gained prominence more recently, driven by a heightened recognition of the profound influence that cancer treatments exert on individuals' sexual lives^44^. The primary aim of this study is to examine the potential influence and association capacity of demographic and characteristics factors on sexual functioning in individuals with CRC. Furthermore, as a secondary outcome, the study seeks to categorize the sexual performance of both male and female CRC patients.

The initial findings of this study indicated the presence of sexual dysfunction in both female and male participants. All female participants exhibited scores within the “poor performance” range across all domains and the total score of the FSFI. Similarly, all male participants fell within the “moderate dysfunction” range in terms of all domains and the total score of the IIEF. These findings align with recent population-based research, which reported a higher prevalence of erectile problems among male survivors of rectal cancer compared to colon cancer and the general population^25^. Various sexual issues, such as difficulties with lubrication, orgasm, and dyspareunia, were also reported. The observed higher prevalence of sexual disorders in patients with colon cancer compared to the general population confirms the impact of colon cancer on sexual function^25,45,46^. Psychological distress has been recognized as a contributing factor to alterations in sexuality, with mood playing a significant role in sexual dissatisfaction and the development of sexual disorders in both illness and everyday life^3,47–49^. Hence, it is plausible to consider that the observed sexual dysfunction in our study may be influenced by psychological factors. The importance of addressing sexual health in individuals diagnosed with colorectal cancer, as underscored by previous research, is further reinforced by the current study.

Another notable finding of our study is the significant potential influence of demographic characteristics on the FSFI total score. Specifically, the following factors demonstrated a substantial potential influence: Colostomy: Participants without a colostomy bag exhibited higher FSFI scores compared to those with a bag. Type of treatment received: Individuals who underwent chemotherapy and chemotherapy-radiotherapy surgery had lower FSFI scores compared to those who underwent chemotherapy and surgery alone. Type of housing: Participants living in their own homes had higher FSFI scores compared to those who were residing in rented accommodations. Occupation: Individuals who were unemployed demonstrated lower FSFI scores compared to those who were employed. Presence of other diseases: Participants without comorbid conditions, besides cancer, exhibited higher FSFI scores compared to those with additional health conditions. Time since the last course of chemotherapy: Participants who had less than 5 weeks since their most recent chemotherapy session displayed lower FSFI scores compared to those who had completed treatment 5–10 weeks ago or more. However, the demographic characteristics of the study participants were found to be association factors for the total score of the International Index of Erectile Function (IIEF). Specifically, the following factors demonstrated significant associations: Education: Participants with educational qualifications below a diploma exhibited lower IIEF scores compared to those with a bachelor's degree or higher. Type of housing: Participants residing in rented accommodations had lower IIEF scores compared to those living in their own homes. Age: Individuals between the ages of 30–40 had higher IIEF scores compared to those aged 40 and above. Type of treatment received: Participants who underwent chemotherapy and chemotherapy-radiotherapy surgery had lower IIEF scores compared to those who received chemotherapy-surgery alone. These findings underscore the significance of these demographic variables in associating sexual function outcomes in individuals with colorectal cancer.

Several studies examining sexual performance in men and women have yielded varying results. Gender appears to play a role in sexual function, with men reporting a higher prevalence of sexual disorders across all areas of sexual functioning^50,51^. While some studies on colorectal cancer (CRC) have reported similar findings, others have found no significant gender differences. It is worth noting that these results differ from those observed in the general population, where sexual dysfunction is typically more prevalent in women^52^. This suggests a potential greater impact of colon cancer on sexual function in men compared to women^53^. Age is consistently identified as a risk factor for sexual disorders, with older patients being more susceptible to experiencing erectile or ejaculation disorders, vaginal dryness, and pain. This trend aligns with observations in the general population, where sexual dysfunctions tend to increase with age, independent of any underlying pathology^51,54,55^. Notably, chemotherapy does not appear to significantly affect sexual function, a finding consistent with existing literature. However, it is important to consider potential memory bias in studies conducted many years after treatment. Future research should aim to investigate the effects of chemotherapy on sexual function during and in the months following treatment, focusing on a homogeneous sample with standardized treatment timelines^56–58^. Moreover, studies assessing the impact of ostomy on sexual life often fail to differentiate between colon and rectal cancers. While a stoma can indeed affect sexual function, factors specific to rectal cancers, such as invasive surgery and radiotherapy, may also contribute to this alteration. These confounding factors may mask the precise impact of a stoma on sexual functioning. Therefore, it is imperative for future research to specifically address this issue in the context of colon cancer^59–61^.

The observed similarities and differences between our study and previous research may stem from various factors, such as variations in participant characteristics, study design, sample sizes, measurement instruments, cultural and societal contexts, healthcare systems, and availability of supportive care services. Furthermore, the timing of data collection in relation to the cancer treatment timeline can impact reported sexual function outcomes. Given the complexity of sexual function and the diverse nature of the colorectal cancer population, it is essential to approach the interpretation and comparison of findings with caution.

### Limitations of this study

The present study acknowledges several limitations that warrant consideration. Firstly, the reliance on self-reported measures introduces the potential for biases and recall inaccuracies, which may impact the validity of the findings. Secondly, the utilization of a cross-sectional design precludes the establishment of causal relationships and temporal sequences. Employing a longitudinal design would offer more robust evidence and enable the examination of changes over time. Additionally, the study's focus on a specific population of colorectal cancer patients limits the generalizability of the findings to other cancer types and broader populations. Furthermore, important factors such as psychological variables and treatment modalities were not explored, despite their potential influence on sexual function. Moreover, the failure to differentiate between colon and rectal cancer represents a significant limitation. These distinct cancer types have differential effects on sexual dysfunction, and analyzing them separately would yield more specific and meaningful insights. Lastly, the absence of pre-treatment assessment of sexual function is a notable limitation. Evaluating sexual function prior to treatment would have provided a baseline measure and enhanced our understanding of the impact of cancer and interventions.

### Recommendations for more studies

Based on the findings and limitations of this study, several recommendations can be made for future research. Firstly, future studies should consider employing a longitudinal design to establish causal relationships and examine changes in sexual function over time. This would provide more robust evidence and enhance our understanding of the long-term effects of cancer and its treatments on sexual function. Secondly, it is important to explore the influence of psychological variables and treatment modalities on sexual function in cancer patients. Investigating factors such as anxiety, depression, and specific treatment regimens can contribute to a more comprehensive understanding of the complexities surrounding sexual dysfunction in this population. Furthermore, future studies should aim to differentiate between colon and rectal cancer and analyze their respective impacts on sexual function separately. This would provide more specific insights and enable tailored interventions for patients based on their cancer type. Additionally, incorporating objective measures of sexual function, such as clinical assessments or physiological measurements, alongside self-reported measures, can enhance the validity and reliability of the findings. Moreover, it is recommended to include a pre-treatment assessment of sexual function in future studies. This would establish a baseline measure and allow for a better understanding of the impact of cancer and its treatments on sexual function. Lastly, expanding the research to include a more diverse sample, including different cancer types and populations, would enhance the generalizability of the findings. This could involve collaboration with multiple research centers or conducting multi-center studies. By addressing these recommendations, future research can contribute to a more comprehensive understanding of sexual dysfunction in cancer patients and guide the development of interventions to improve their sexual well-being and overall quality of life.

## Conclusion

In conclusion, this cross-sectional study illuminates the significant contributing factors associated with sexual dysfunction in colorectal cancer patients in Iran. These findings underscore the importance of addressing sexual health concerns in this population, providing valuable insights for healthcare providers and policymakers. The study aims to guide them in developing strategies that address the unique challenges faced by colorectal cancer patients in the realm of sexual health.

### Supplementary Information

Supplementary Information 1.

Supplementary Information 2.

Supplementary Information 3.

## Data Availability

All data in SPSS file has been uploaded to supplementary material section. Data of this manuscript has been gathered through questionnaires by patients themselves the questioner was actively present when the questionnaires were filled and provided the necessary guidance in filling the questionnaires by the patients. The data sets used and analyzed for the current study are available upon reasonable request of the corresponding author Dr. Mohammadhassan Sahebihagh (sahebihagh@yahoo.com).

## References

[1] Kaiser, J. *Cancer. Encyclopedia of Evolutionary Psychological Science*, 871–874 (2021).

[2] Siegel RL, Miller KD, Fuchs HE, Jemal A (2021). Cancer statistics, 2021. CA Cancer J. Clin..

[3] Stulz A, Lamore K, Montalescot L, Favez N, Flahault C (2020). Sexual health in colon cancer patients: A systematic review. Psychooncology.

[4] Morgan E, Arnold M, Gini A, Lorenzoni V, Cabasag C, Laversanne M (2023). Global burden of colorectal cancer in 2020 and 2040: Incidence and mortality estimates from GLOBOCAN. Gut.

[5] Ostadghaderi M, Hanafi Bojd A, Nematollahi S, Holakoui-Naeini K (2021). Spatial analysis of factors affecting colorectal cancer using the model of geographical weight regression in Iran. Iran. J. Epidemiol..

[6] Abbastabar H, Roustazadeh A, Alizadeh A, Hamidifard P, Valipour M, Valipour AA (2015). Relationships of colorectal cancer with dietary factors and public health indicators: An ecological study. Asian Pac. J. Cancer Prev..

[7] Khademi IKH, Tehnizi MAH, Shafizad S (2021). The effect of self-care education program on self-efficacy and quality of life of patients with colorectal cancer undergoing chemotherapy. J. Prev. Med..

[8] Collatuzzo G, Seyyedsalehi MS, Rezaeianzadeh A, Marzban M, Rashidian H, Hadji M (2022). Consumption of yoghurt and other dairy products and risk of colorectal cancer in Iran: The IROPICAN Study. Nutrients.

[9] Mohammadi E, Aminorroaya A, Fattahi N, Azadnajafabad S, Rezaei N, Farzi Y (2021). Epidemiologic pattern of cancers in Iran; Current knowledge and future perspective. J. Diabetes Metab. Disord..

[10] Facchin F, Buggio L, Vercellini P, Frassineti A, Beltrami S, Saita E (2021). Quality of intimate relationships, dyadic coping, and psychological health in women with endometriosis: Results from an online survey. J. Psychosom. Res..

[11] Kayser K, Acquati C, Reese JB, Mark K, Wittmann D, Karam E (2018). A systematic review of dyadic studies examining relationship quality in couples facing colorectal cancer together. Psychooncology.

[12] World Health Organization (2001). Guide to WHO Documents Concerning Adolescent Health and Development.

[13] Bradley L, Noble N, Hendricks B (2023). DSM-5-TR: Salient changes. Fam. J..

[14] Bahnsen MK, Graugaard C, Andersson M, Andresen JB, Frisch M (2022). Physical and mental health problems and their associations with inter-personal sexual inactivity and sexual dysfunctions in Denmark: Baseline assessment in a national cohort study. J. Sex. Med..

[15] Masoudi M, Maasoumi R, Bragazzi NL (2022). Effects of the COVID-19 pandemic on sexual functioning and activity: A systematic review and meta-analysis. BMC Public Health.

[16] Benedict C, Fisher S, Kumar D, Pollom E, Schapira L, Kurian AW (2022). Examining associations among sexual health, unmet care needs, and distress in breast and gynecologic cancer survivors. Seminars in oncology nursing.

[17] Acquati C, Wittmann D, Roth M, Rosen A, Carr LC, Gresham Z (2023). Sexual health outcomes of adolescent and young adult colorectal cancer survivors and their partners: Protocol of a dyadic mixed methods study. JMIR Res. Protoc..

[18] Gatien C (2021). L'association entre la qualité de la relation conjugale et les symptômes de dépression et d'anxiété chez les personnes aux prises avec une douleur chronique.

[19] Peleg Nesher S, Luria M, Shachar E, Percik R, Shoshany O, Wolf I (2022). Sexual dysfunction among adolescent and young adult cancer patients: Diagnostic and therapeutic approach. Curr. Opin. Support. Palliat. Care.

[20] Stulz A, Favez N, Flahault C (2022). Emotional and sexual adaptation to colon cancer: Perceptual congruence of dyadic coping among couples. Front. Psychol..

[21] Sprangers M, Taal B, Aaronson N, Te Velde A (1995). Quality of life in colorectal cancer. Dis. Colon Rectum.

[22] Sprangers M, Taal B, Aaronson N, Te Velde A (1995). Quality of life in colorectal cancer: Stoma vs. nonstoma patients. Dis. Colon Rectum.

[23] Grant M, McMullen CK, Altschuler A, Mohler MJ, Hornbrook MC, Herrinton LJ (2011). Gender differences in quality of life among long-term colorectal cancer survivors with ostomies. Oncology Nursing Forum.

[24] Reese J, Finan P, Haythornthwaite J, Kadan M, Regan K, Herman J (2014). Gastrointestinal ostomies and sexual outcomes: A comparison of colorectal cancer patients by ostomy status. Support. Care Cancer.

[25] Den Oudsten B, Traa M, Thong M, Martijn H, De Hingh I, Bosscha K (2012). Higher prevalence of sexual dysfunction in colon and rectal cancer survivors compared with the normative population: A population-based study. Eur. J. Cancer.

[26] Fish D, Temple LK (2014). Functional consequences of colorectal cancer management. Surg. Oncol. Clin..

[27] Wieldraaijer T, Duineveld L, Van Asselt K, van Geloven A, Bemelman W, van Weert H (2017). Follow-up of colon cancer patients; causes of distress and need for supportive care: Results from the ICARE Cohort Study. Eur. J. Surg. Oncol. (EJSO).

[28] Behroozian T, Fatima S, Finkelstein S, Kanee L, Bonomo P, Wolf JR (2022). Current quality of life assessment tools may not fully address dermatological adverse events from anti-cancer therapies. Support. Care Cancer.

[29] Al-Habsi Z, Al-Noumani H, Al HI (2022). Determinants of health-related quality of life among Omanis hospitalized patients with cancer: A cross-sectional study. Qual. Life Res..

[30] Xu J, Xue B, Li L, Qiao J, Redding SR, Ouyang YQ (2023). Psychological interventions for sexual function and satisfaction of women with breast cancer: A systematic review and meta-analysis. J. Clin. Nurs..

[31] Panzeri M, Dadomo H, Ronconi L, Fontanesi L (2021). Validation of the sexual inhibition/sexual excitation scales (SIS/SES) in Italy: Assessing gender and age differences of sexual functioning. Arch. Sex. Behav..

[32] Abd El Salam S, Hassan H, Kamal K, Ali R (2021). Sexual dysfunction of women’s associated with cervical cancer. J. Appl. Health Sci. Med..

[33] Prasad S (2023). Menopause in ethnic minority women. Post Reprod. Health.

[34] Nik Jaafar NR, Selamat Din SH, Mohamed Saini S, Ahmad SN, Midin M, Sidi H (2014). Clinical depression while caring for loved ones with breast cancer. Compr. Psychiatry.

[35] Green SB (1991). How many subjects does it take to do a regression analysis. Multivar. Behav. Res..

[36] Mann Whitney U test calculator [Internet]. http://www.statskingdom.com/170median_mann_whitney.html (2017).

[37] Rosen-Grandon JR, Myers JE, Hattie JA (2004). The relationship between marital characteristics, marital interaction processes, and marital satisfaction. J. Couns. Dev..

[38] Mohammadi, K., Heydari, M. & Faghihzadeh, S. Validity of the Persian version of the female sexual function index-FSFI scale as an indicator of female sexual function.

[39] Asil J (2017). The effect of sexual intelligence training on sexual performance of couples. J. Shahrekord Univ. Med. Sci..

[40] Mmohammadi Z, Ghaffari S (2009). Sexual dysfunction and its relationship with quality of life in female cancer patients. Iran. J. Obstet. Gynecol. Infertil..

[41] Rosen RC, Riley A, Wagner G, Osterloh IH, Kirkpatrick J, Mishra A (1997). The International Index Of Erectile Function (IIEF): A multidimensional scale for assessment of erectile dysfunction. Urology.

[42] Pakpour AH, Zeidi IM, Yekaninejad MS, Burri A (2014). Validation of a translated and culturally adapted Iranian version of the international index of erectile function. J. Sex Marital Therapy.

[43] Grenier B (1995). Mesurer l'incommensurable? La mesure de la qualité de la vie. La Revue de médecine interne.

[44] Reese JB, Keefe FJ, Somers TJ, Abernethy AP (2010). Coping with sexual concerns after cancer: The use of flexible coping. Support. Care Cancer.

[45] Buvat J, Glasser D, Neves RCS, Duarte FG, Gingell C, Moreira ED (2009). Sexual problems and associated help-seeking behavior patterns: Results of a population-based survey in France. Int. J. Urol..

[46] Ballering AV, Olde Hartman TC, Verheij R, Rosmalen JG (2023). Sex and gender differences in primary care help-seeking for common somatic symptoms: A longitudinal study. Scand. J. Prim. Health Care.

[47] Mües HM, Kirchheiner K, Grabovac I (2023). Promotion of Sex in Older Adults. Sexual Behaviour and Health in Older Adults.

[48] Lewis RW, Fugl-Meyer KS, Bosch R, Fugl-Meyer AR, Laumann EO, Lizza E (2004). Epidemiology/risk factors of sexual dysfunction. J. Sex. Med..

[49] Mohamad Muhit AM, Sy-Cherng Woon L, Nik Mhd Nor NS, Sidi H, Mohd Kalok AH, Kampan NC (2022). Sexual dysfunction among gynaecological cancer survivors: A descriptive cross-sectional study in Malaysia. Int. J. Environ. Res. Public Health.

[50] Lange M, Marijnen C, Maas C, Putter H, Rutten H, Stiggelbout A (2009). Risk factors for sexual dysfunction after rectal cancer treatment. Eur. J. Cancer.

[51] Han CJ, Yang GS, Syrjala K (2020). Symptom experiences in colorectal cancer survivors after cancer treatments: A systematic review and meta-analysis. Cancer Nurs..

[52] Gorman JR, Arthur EK (2022). Reproductive and Sexual Health Concerns for Cancer Survivors.

[53] Nicolosi A, Buvat J, Glasser DB, Hartmann U, Laumann EO, Gingell C (2006). Sexual behaviour, sexual dysfunctions and related help seeking patterns in middle-aged and elderly Europeans: The global study of sexual attitudes and behaviors. World J. Urol..

[54] Thyø A, Elfeki H, Laurberg S, Emmertsen K (2019). Female sexual problems after treatment for colorectal cancer—A population-based study. Colorectal Dis..

[55] Magaji BA, Moy FM, Law CW, Sii HL, Roslani AC (2019). Pattern of health-related quality of life and its association among patients with colorectal cancer. Asian Pac. J. Cancer Care.

[56] Chokshi A, Belekar DM, Chokshi S (2023). Sexual health of colorectal cancer patients—A systematic review. Indian J. Surg..

[57] Attaallah W, Ertekin SC, Yegen C (2018). Prospective study of sexual dysfunction after proctectomy for rectal cancer. Asian J. Surg..

[58] Almont T, Couteau C, Etienne H, Bondil P, Guimbaud R, Schover L (2018). Sexual health and needs for sexology care in digestive cancer patients undergoing chemotherapy: A 4-month cross-sectional study in a French University Hospital. Support. Care Cancer.

[59] Dahouri A, Sahebihagh MH, Gilani N (2023). Comparison of sexual function of people with colorectal cancer with and without colostomy bag in Iran: A comparative cross-sectional study. Sci. Rep..

[60] García-Rodríguez MT, Barreiro-Trillo A, Seijo-Bestilleiro R, González-Martin C (2021). Sexual dysfunction in ostomized patients: A systematized review. Healthcare.

[61] Bahayi K, Attaallah W, Yardımcı S, Bulut H, Özten E (2018). Depression, anxiety, sexual dysfunction and quality of life in patients with ileostomy or colostomy. Turk. J. Colorectal Dis..

